# Hip abduction weakness in elite junior footballers is common but easy to correct quickly: a prospective sports team cohort based study

**DOI:** 10.1186/1758-2555-4-37

**Published:** 2012-10-02

**Authors:** Hamish R Osborne, John F Quinlan, Garry T Allison

**Affiliations:** 1 Department of Medicine, Dunedin School of Medicine, University of Otago, Great King St, Dunedin, New Zealand; 2 School of Physiotherapy, Curtin University, Kent St, Bentley, Western Australia, Australia

## Abstract

**Background:**

Hip abduction weakness has never been documented on a population basis as a common finding in a healthy group of athletes and would not normally be found in an elite adolescent athlete. This study aimed to show that hip abduction weakness not only occurs in this group but also is common and easy to correct with an unsupervised home based program.

**Methods:**

A prospective sports team cohort based study was performed with thirty elite adolescent under-17 Australian Rules Footballers in the Australian Institute of Sport/Australian Football League Under-17 training academy. The players had their hip abduction performance assessed and were then instructed in a hip abduction muscle training exercise. This was performed on a daily basis for two months and then they were reassessed.

**Results:**

The results showed 14 of 28 athletes who completed the protocol had marked weakness or a side-to-side difference of more than 25% at baseline.

Two months later ten players recorded an improvement of ≥ 80% in their recorded scores. The mean muscle performance on the right side improved from 151 Newton (N) to 202 N (p<0.001) while on the left, the recorded results improved from 158 N to 223 N (p<0.001).

**Conclusions:**

The baseline values show widespread profound deficiencies in hip abduction performance not previously reported. Very large performance increases can be achieved, unsupervised, in a short period of time to potentially allow large clinically significant gains. This assessment should be an integral part of preparticipation screening and assessed in those with lower limb injuries. This particular exercise should be used clinically and more research is needed to determine its injury prevention and performance enhancement implications.

## Background

The gluteus medius (GMed) muscle originates from the lateral aspect of the ilium between the posterior and anterior gluteal lines and inserts into the lateral aspect of the greater trochanter. It is mainly an abductor of the hip but its posterior part can effect external rotation of the joint. In terms of locomotion it is very active immediately prior to heel strike [1] – thus being very important in pelvi-femoral control at heel strike and immediately after. It is also very active during the single support period of the stance phase and prevents falling of the body to the unsupported side. Clinically, inadequate function in the GMed can be detected by means of the Trendelenburg test as first described by Freidrich Trendelenburg in 1897 and more recently elucidated by Hardcastle [2]. In the latter paper, it was noted that Medical Research Council (MRC) muscle power grades [3] of equal or less than 4 (muscle strength is reduced but muscle contraction can still move joint against resistance) would lead to a positive fatigue sign between 0 and 25 seconds.

Tensor fascia lata and gluteus maximus are the other two important hip abductors. They attach more anteriorly and more posteriorly respectively to the pelvis than GMed and create abduction at the hip by pulling on the tibia via the iliotibial band. While both are active prior to heel strike it’s likely that gluteus medius is more important than tensor fascia lata and gluteus maximus prior to heel strike and the reverse after heel strike based on peak and mean results from electromyographic studies [4].

Recent literature has focused on the dynamic neuromuscular control of the trunk, hip and femur over the planted leg [5] and the role the gluteus medius plays in this. Weakness of the GMed leads to increased femoral internal rotation [6], which in turn leads to an increased knee valgus angle [7]. This has led to an association between GMed weakness and patello-femoral pain syndrome [1,8]. In addition, the gender differences noted in GMed strength with women having weaker hip abduction and external rotation [9], have led authors to question a possible association with the increased risk of anterior cruciate ligament ruptures seen in female athletes [10,11]. Decreased control of the femoro-pelvic rotation attributed to the GMed muscle performance has also been associated with osteitis pubis [12,13]. This condition has been described in many sports that involve repetitive kicking, side-to-side movement or twisting [14-16]. In particular, it appears to be of significantly high incidence in Australian Rules football [12,17]. Further sequelae of a weak GMed include iliotibial band syndrome [6] which accounts for 1.6-12% of all running related injuries [18-20]. Fredericson’s study [6] showed significant improvement in iliotibial band syndrome symptoms with hip abduction strengthening. Greater trochanteric pain syndrome, which has been described as being secondary to a weak GMed [21] has been shown to account for up to 20% of referrals to a spinal outpatient service as well as accounting for hip pain presenting as low back or buttock pain [21].

This study set out to highlight the very poor hip abduction in an elite group of under-age Australian Rules Footballers using a previously unreported test. It also aimed to demonstrate the significant improvements that can be attained over a short period of time with focused and yet unsupervised rehabilitation.

The research questions were:

1. How common and how significant are hip abduction deficiencies in elite junior footballers?

2. Are these deficiencies easily corrected with an unsupervised home based exercise program?

## Methods

Each year, the Australian Institute of Sport (AIS) and Australian Football League (AFL) invites the top 30 under-17 year old Australian Rules Football players to a year-long non-residential academy program. During this period, four residential training camps are held during which time all players are physically assessed. At the entry point to the study none had injuries or pain that prevented them from completing the full battery of AIS/AFL draft camp fitness and agility tests.

**Figure 1:**
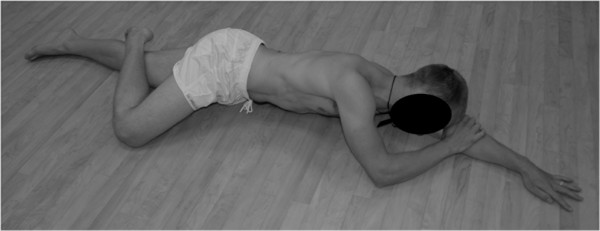
**Initial position for the hip abduction exercise.** The player lies on their side with their head on the underneath arm and the elbow of the top arm on the floor level with the chin in front of their face.

**Figure 2:**
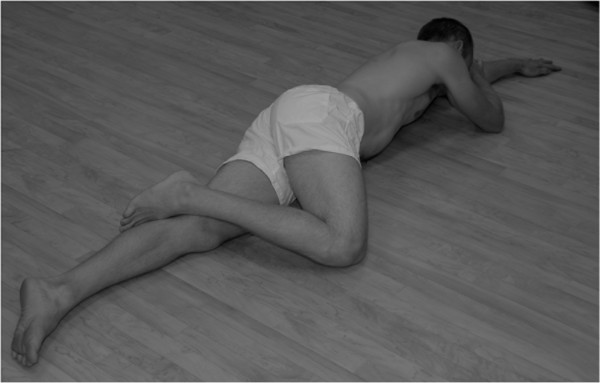
**The bottom leg is kept in line with the trunk and the top leg is flexed at the hip with the toes placed behind the lower knee.** The pelvis is rolled forward so that the slope of the pelvis matches the slope of the shoulders.

**Figure 3:**
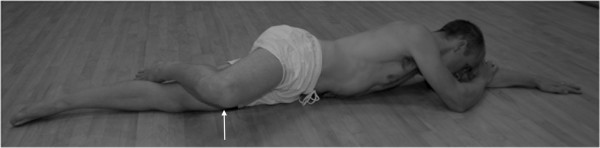
With a flexed knee, the player lifts the knee 10cm off the floor (maintaining trunk alignment), lowers the knee to the floor and then relaxes each time.

The team sports physician individually instructed each player in the same specific hip abduction exercise. The specific exercise involved had the player lie on their side with their head on the underneath arm and the elbow of the top arm on the floor in front of their face (Figure 1). The bottom leg is kept in line with the trunk and the top leg flexed at the hip with the toes placed behind the other knee (Figure 2). They rotated their hips, by sliding the knee away from the body, so that their pelvis was on the same slope as the shoulders. Then whilst maintaining pelvic positioning relative to the shoulders, they abducted their hip lifting the knee 10cm from the floor (Figure 3) before then lowering the knee to the floor and relaxing the abducting muscles. The athletes were video-taped performing the exercises. Each athlete was provided with personalized feedback and correction of the exercise and a copy of their video.

The following day when the athletes had been able to practice a small number of the exercises for familiarisation all athletes had their hip abduction performance assessed using a hand held dynamometer and the best of three repeat measurements recorded. The tests were done on each occasion when no other exercise had been performed on that day to avoid any other fatigue factors. Each player was prescribed 100 repetitions per side per day. The exercise was then performed by the athlete in their homes across Australia unsupervised for the two months duration between the team camps. At the second camp repeated measurements were again performed using an identical protocol. The choice of this position for testing and training and the number of repetitions for the exercise was based on the clinical practice of the team sports physician.

A small reliability study was conducted on the clinical assessments for within and between days performance on 10 athletes. The within day 95% confidence limits were 23% and 20% with a between days ICC of 0.67 (95% CI. 0.12 – 0.91). Therefore, a threshold of 25% was defined as the minimal detectable difference (95% confidence limits) for this measurement technique. Due to the technique and study cohort of this clinical test it is unclear what magnitude of change would be considered to be the minimal clinically significant difference.

All players and their parents/guardians signed pre-academy agreements that included permission for collection of data for the purpose of manuscript preparation in accordance with AIS policy at the time. In addition, the AIS/AFL scholarship contracts specifically allow for research to be undertaken and data collected during the scholarship to be published as per clauses 5.2 and 5.3. Furthermore, this project was formally assessed and approved by the Ethics Committee of the AIS prior to manuscript submission (reference no. 20090605).

All thirty athletes from and AIS/AFL Academy intake were enrolled in the study. The team sports physician performed all testing and exercise prescription. The testing was performed at the Australian Institute of Sport in Canberra, Australia. The exercises were performed by the athletes in their own centres in the two months between camps. The phase of the season during which the study was conducted was immediately post finals series an as such was a period of relative rest with cross training and some individual rehabilitation programs.

Primary outcome: All 30 players had their hip abduction performance measured by the team sports physician with 10 years experience in the assessment of junior elite athletes. A hand held dynamometer, Commander PowerTrack II™ muscle tester (JTECH Medical, Salt Lake City, Utah, USA), was used. The hand held dynamometer was held firmly on the lateral aspect of the athletes knee in the position used for their home based exercise and the athlete asked to lift their knee with maximal exertion for approximately three seconds (a make test). Each side was evaluated in triplicate and the highest score recorded. It was noted that no reference range exists for such performance testing.

At the second testing, 2 months later, 2 players were unavailable. Their initial data was discarded and results were calculated from the remaining 28 players. All players again had their hip abduction performance measured by the original assessor using the Commander PowerTrack II™ muscle tester and an identical protocol. Similar to the original time point, the scores were measured in duplicate and the highest result was recorded.

To help make clinical correlations to bedside non hand held dynamometry strength testing the strength of the authors (HRO) index finger was arbitrarily defined as the cut off between MRC grade 4 and 5 strength (“active movement, against gravity and resistance” [3,22]). The cut off was set at 130N – the maximum strength of the authors (HRO) right index finger as measured pushing straight down on the hand held dynamometer on a desktop i.e. the athletes were unable to overcome the strength of the examiners index finger.

All statistical analysis was performed using paired T testing from the Microsoft Excel program (Microsoft Corporation, Seattle WA, USA). Statistical significance was taken for all values of p≤0.05.

## Results

All 30 athletes were instructed in the exercise and had baseline tests recorded. At the second testing, 2 months later, 2 players were unavailable. One player was medically unwell and did not attend the camp. The second player had suffered a lower limb fracture. Their initial data was discarded and results were calculated from the remaining 28 players.

The mean (mean +/− standard deviation) age of the study cohort was 16.7+/−0.3 years (range: 16.0 – 17.6 years). Their mean height was 189 cm (SD=5.8) (range: 179 – 199cm) and the mean weight of the group was 79 kg (SD=4.8) (range: 69 – 89 kg).

**Figure 4:**
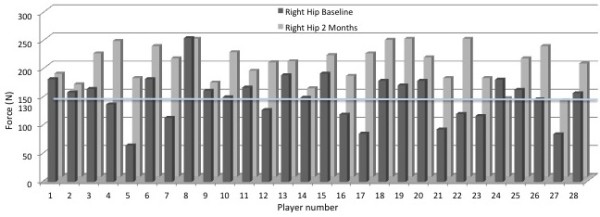
**Graph showing results of original and retesting of the right hip abduction for each individual player.** The 130N line represents the cut off line for grade 4/5 strength.

**Figure 5:**
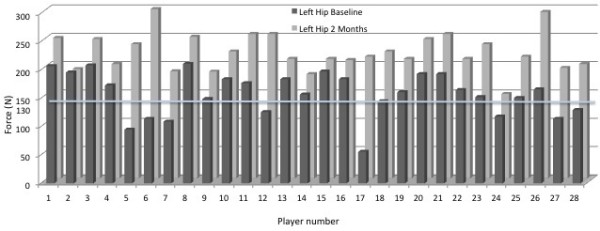
**Graph showing results of original and retesting of the left hip abduction for each individual player.** The 130N line represents the cut off line for grade 4/5 strength.

At initial testing, the mean muscle performance for right hip abduction was 151 Newton’s (N) (SD=40.7) (range: 65 – 256 N) while the left side had a mean of 158 N (SD=38.7) (range: 56 – 211 N). The mean percentage difference between the 2 sides was 23% (SD=21.8). This was not statistically significant (p=0.318). Of the 28 athletes, 12 recorded hip abduction performance on one or more legs of less than 130 N (Figures 4 and Figure 5).

Although on a group basis there was no statistically difference between sides, when a threshold was set at 25%, 11 athletes exceeded this with a mean side-to-side difference of 48% (SD=23.1).

Following the 2 months of hip abduction exercises repeat tests were performed. The mean muscle performance on the right was 202 N (SD=32.4) (range: 136 – 246 N) (Figure 4). The mean tested performance on the left side rose to 224 N (SD=33.1) (range: 149 – 299 N) (Figure 5). The mean percentage difference between the sides reduced to 17% (SD=13.6) although statistically this wasn’t different from baseline (p=0.131). Highly significant improvements (p<0.001) were found on each side when the second tests were compared with the original observations (Figures 4 and Figure 5).

In respect of the players who showed deterioration in scores between the 2 time intervals, 2 had drops of only 4% and 2%. These players improved their opposite sides by 18% and 3% respectively. The final player recorded a drop of 23% between readings while improving his contralateral side by 26%. On further questioning, all 3 players involved admitted to sub-optimal adherence to the exercise regimen.

At the other end of the spectrum, 14 players recorded improvements of greater than or equal to 50% in their muscle retest scores. Seven of these demonstrated these improvements on both sides. In 11 cases the right improved by ≥50% by a mean improvement of 91% (SD=41.8). In 10 cases, the left side improved by a mean improvement of 82% (SD=78.0).

Again with the threshold set at 25%, at retesting only 6 players recorded a difference of greater than 25% between sides and the mean side-to-side difference had dropped to 34% (SD=6.5).

## Discussion

The baseline measurements showed that 12 of the 28 athletes had hip abduction strength of MRC grade 4. In total, at baseline, 14 out of 28 athletes had MRC grade 4 strength or greater than a 25% side-to-side difference in performance. These profound lopsided performance and/or pure hip abduction deficiencies have never been previously reported in asymptomatic athletes.

After two months of the exercise all had improved to grade 5. In fact some athletes doubled or even tripled their baseline scores. These observed changes were large compared to the threshold of detection set with the small pilot study. These improvements should reduce the chance that athletes still had Trendelenburg gait [2] and increased valgus knee moments with landing manoeuvres [23] but this was not tested in this study.

An unsupervised exercise program in an adolescent population can have significant compliance problems. This exercise without knowing exact compliance details resulted in no athletes continuing to have fundamental hip abduction deficiencies.

Fredericson in his review of iliotibial band syndrome in athletes [6] makes reference to the long held belief that athletes with a greater than 10% disparity between muscle groups are more prone to injury. In our cohort, 11 players originally had greater than 25%. In fact the side-to-side differences in this subgroup were huge with a mean difference between the sides of 48%. While it was noted at retesting that 6 players still recorded difference of greater than 25% between sides, the mean difference had dropped to 34%. With close supervision it would be reasonable to anticipate achieving no athletes with a greater than 10% side to side difference although measuring this accurately in the clinical setting may prove difficult.

These results would suggest that if the 10% disparity is valid, and Trendelenburg positive gait is shown to be a risk factor for lower limb injury, the group’s injury profile should be diminished following their relatively short period of focused rehabilitation and into the future with continued hip abduction training.

The exercise used in this intervention has not been previously studied. There are no normative values for hip abduction performance in this position. Pure hip abduction performance has been measured in a smaller group of slightly older elite association footballers [24]. A mean side-to-side difference of 14% in these athletes was noted for hip torque but not noted in controls. No fundamental strength deficiencies were reported. Hip abduction deficits have been shown in a group of injured runners but only on their injured side [25]. Had these athletes been tested in the position used in our study it’s possible that more common and profound deficits would have been noted in injured and non-injured limbs and in the control group.

This exercise is used clinically by the teams sports physician (HRO) to treat a number of lower limb biomechanically related injuries as anecdotally similar deficiencies and subsequent gains are seen across the spectrum of injured athletes and non athletes alike. This exercise is used by the clinician for several reasons and because the literature fails to support any one exercise over another a process of reasoned assumption has been made. If injuries such as ilio tibial band syndrome and greater trochanteric pain syndrome are related to GMed weakness, how is it that when patients are performance tested in the clinical setting in pure abduction, strength is almost always grade 5 yet in the position of the exercise used in this study performance deficiencies noted? It is therefore logical to strengthen in the position of weakness. In the clinical scenario it has been observed using manual palpation of the muscles that the position used in this study minimizes tensor fascia lata and gluteus maximus contraction, making it likely that gluteus medius is the dominant muscle however this needs further laboratory based research.

Anatomically GMed is the largest abductor of the femur from the pelvis, tensor fascia lata and gluteus maximus causing leg abduction via the fascia lata to the tibia. It has been shown that in males peak contraction of GMed compared with gluteus maximus prior to heel strike during single leg landings expressed as percentage of maximum voluntary isometric contraction is the same (48% compared to 47% respectively) [4] but the mean contraction of GMed compared with gluteus maximus similarly expressed is much greater in GMed (26%) compared to gluteus maximus (16%) [4]. This information would suggest that not only is GMed most ideally positioned anatomically but it is the key controller of lateral pelvic control at heel strike If GMed is fundamentally weak then coronal plane control of the pelvis at heel strike must be compromised i.e. Trendelenburg gait type variants [26]. Given that this proximal control is required for distal strength and co-ordination it follows that heel strike with poor GMed strength is the beginning of the abnormal movement patterns that lead to injury related to landing and impact whether acute or repeated.

If GMed is the key controller of pelvic tilt at heel strike then this would go some way to explaining the very large increases in performance seen in this study that would not normally be expected from a low load, high repetition activity. The weaknesses observed in this study at baseline are commonly seen clinically, in fact it’s not that uncommon to observe a grade 3 deficiency i.e. a patient unable to lift their knee from the floor. GMed must have a threshold of performance below which it cannot participate in a useful way in the normal gait cycle, otherwise it would not remain fundamentally weak. This is observed clinically as many patients with overload injuries related to GMed weakness (e.g. iliotibial band syndrome, greater trochanteric pain syndrome) have significant reduction in their pain by approximately four weeks of rehabilitation using this exercise. Once this currently unknown threshold (estimated to occur clinically after 4 weeks of exercises) is reached the GMed is then likely starting to actively participate in normal gait cycle and therefore the number of repetitions performed over the next month would increase exponentially to perhaps 5000 repetitions daily (its common for fit health people to take 10000 steps per day). Moreover GMed would no longer just be lifting the weight of the leg performing the exercise but actually supporting most of the body weight at heel strike. This would account for the very large increases in performance observed. It would be prudent with further research to do serial testing to observe if there is a threshold of performance after which there is a significant increase in performance increments compared to below this threshold.

Risk of injury is associated with overuse, a phenomenon which is relatively high in AFL [27]. Intuitively, it would be assumed that weaker muscle groups are prone to overuse injuries earlier. Overuse is associated with sports related chronic groin pain [17] which is the third commonest reason for missed club games in the AFL [28]. In particular, players in this code are associated with a relatively high incidence of osteitis pubis which has been associated with a weakness in GMed [12,13].

Other codes of football show similar injury patterns. Bathgate’s review of Australian rugby union injuries [29] showed that injuries to the knee accounted for the largest proportion of severe injuries (25%). Of these 34% occurred in non-tackling situations. Although this is somewhat lower than in AFL [30] where 76% of anterior cruciate ligament injures occur in non tackling situations it still represents an area where GMed weakness could potentially be contributing to a very significant problem. The relatively high level of injuries seen in association football especially to the lower limb again raises the issue of overall proximal control of pelvis and centre of mass over the planted leg [5].

Current research trends into anterior cruciate ligament ruptures include the difference in landing techniques between males and females [31]. There is focus on the technical problems of landing with interest in quadriceps dominant landings, ankle dorsiflexion angles and also in prevention strategies including GMed strengthening and landing practice. However it has yet to be published that GMed weakness exists in those who rupture their anterior cruciate. This study moves towards finding that missing link, that GMed weakness is common and would help to account for the high level of injuries linked to its weakness despite the weakness being assumed but not previously shown.

This would especially be true for females where the broader female pelvis makes pelvi femoral control more difficult and leads to significantly more problems with injuries related to GMed weakness such as patello femoral syndrome and greater trochanteric pain syndrome (where increased knee valgus angles and GMed weakness have clear associations). It would be expected to find GMed deficiencies similar to those found in this study in elite female athletes. This is an area where future research should be focused.

This study has some obvious limitations. There was no control group. The exercise technique has not been shown to be an exercise that focuses on GMed over other hip abductors in a laboratory based study. Further anatomical and electromyographic research should be done on this particular exercise to determine that in this position maximum recruitment of GMed occurs with minimal recruitment of tensor fascia lata and gluteus maximus, in comparison with other hip abduction exercises. Other hip abduction exercises are described often in the literature but generally the authors are focused on hip abduction strength rather than individual muscle performance deficiencies [32]. There is a chance that the exercise does not primarily use GMed, but the results of the study remain significant and the exercise remains anecdotally a powerful clinical tool.

The lack of any weight lifted other than that of the athletes own leg can be criticized but a lower limb is of sizable weight. Hand held dynamometry is not the gold standard for strength testing and certainly isometric testing isn’t as good as laboratory based strength testing. However hand held dynamometry quite closely correlates with laboratory based dynamometry and is a valid clinical tool [32,33].

The athletes were given 24 hours to practice the exercise between it being taught and the hand held dynamometry being performed. We believe this makes it unlikely that the large gains observed in the following two months are likely to be related to a learning phenomenon given the simplicity of the exercise. However the large threshold of detection set by the small pilot study could be improved with tighter testing protocols and strategies to minimise performance variance in the athletes.

However many of these criticisms can be seen as a positive as this study was undertaken at the coalface of elite sport on the sideline of a sporting field using readily available equipment. That such huge deficiencies were found and then corrected in a traditionally difficult age group to obtain compliance and that it was done essentially unsupervised would suggest that these are real results that can be achieved by a clinician in their clinic without the need for expensive equipment and lots of supervision time.

## Conclusions

The relevance of the study to elite athletes is that 24 months after testing, 25 of the 30 test participants are full-time professional AFL players. As such, this group represents a truly elite sample of adolescent sportsmen. These findings raise the question as to the level of hip abduction weakness in athletes of both lower and higher grades, other sports and the non-athletic population when tested using the technique in this study. Many of these footballers were truly skilled athletes, also in other Australian teams e.g. Australian Under 18 Cricket and Basketball teams and many were nationally ranked athletes in track and field, so it’s certainly possible and more than likely that these results are generalizable to other sports.

The results of this study suggest the importance of hip abduction strengthening in elite junior athletes given that there appears to be common and profound side-to-side differences and large performance deficiencies even at this level. This study highlights the relatively large improvements that can be made over a short period of time with appropriately focused rehabilitation. The fact that the muscle performance is so amenable to a high repetition low load training task suggests that there are neuro-motor control elements that are fundamental factors contributing to this change in performance. It is likely that interactive elements of muscle capacity and control of the lumbo-pelvic motion during common movements undertaken in kicking sports contributes to the prevalence of injuries associated with the GMed muscle performance deficiencies.

## Competing interests

There are no competing interests.

## Authors’ contributions

HRO conceived and designed the study, collected the data and assisted with writing. JFQ performed analyzed the data and drafted the manuscript. GTA assisted with conception of the study and assisted in revising the manuscript. All authors read and approved the final manuscript.
